# Transformation of BCC and B2 High Temperature Phases to HCP and Orthorhombic Structures in the Ti-Al-Nb System. Part II: Experimental TEM Study of Microstructures

**DOI:** 10.6028/jres.098.039

**Published:** 1993

**Authors:** L. A. Bendersky, W. J. Boettinger

**Affiliations:** National Institute of Standards and Technology, Gaithersburg, MD 20899-0001

**Keywords:** domain interfaces, phase transformations, thermodynamics, Ti-Al-Nb phases, transmission electron microscopy

## Abstract

Possible transformation paths that involve no long range diffusion and their corresponding microstructural details were predicted by Bendersky, Roytburd, and Boettinger [J. Res. Natl. Inst. Stand. Technol. **98**, 561 (1993)] for Ti-Al-Nb alloys cooled from the high temperature BCC/B2 phase field into close-packed orthorhombic or hexagonal phase fields. These predictions were based on structural and symmetry relations between the known phases. In the present paper experimental TEM results show that two of the predicted transformation paths are indeed followed for different alloy compositions. For Ti-25Al-12.5Nb (at%), the path includes the formation of intermediate hexagonal phases, A3 and DO_19_, and subsequent formation of a metastable domain structure of the low-temperature O phase. For alloys close to Ti-25Al-25Nb (at%), the path involves an intermediate B19 structure and subsequent formation of a translational domain structure of the O phase. The path selection depends on whether B2 order forms in the high temperature cubic phase prior to transformation to the close-packed structure. The paper also analyzes the formation of a two-phase modulated microstructure during long term annealing at 700 °C. The structure forms by congruent ordering of the DO_19_ phase to the O phase, and then reprecipitation of the DO_19_ phase, possibly by a spinodal mechanism. The thermodynamics underlying the path selection and the two-phase formation are also discussed.

## 1. Introduction

Bendersky, Roytburd, and Boettinger [1] have analyzed different phase transformation sequences and corresponding microstructures that may be possible in the course of transformations with no long-range diffusion (partitionless) of a BCC-based high temperature phase to close-packed phases for Ti-Al-Nb alloys. The analysis is based only on the crystallography of the equilibrium low and high temperature phases existing near the (Ti,Nb)3Al binary join. Based on formal maximal group/subgroup relations, possible connections spanning the high and low temperature phases (corresponding to high and low symmetry) have been found. These relations give different sequences involving symmetry-decreasing subgroups and one symmetry increasing-supergroup that might be considered to represent possible transformation paths. The transformation paths involve intermediate transitional structures, possibly metastable or unstable.

Assuming that the formal transformation steps actually occur as phase transitions, different transformation paths will result in different sequences of domain formation, i.e., in different final microstructures. The final single phase microstructures can be distinguished by the type and hierarchy of domain interfaces. Analysis of the interfaces is essential for understanding the transformation path, and is the major subject of this paper.

Transformation paths, derived from group/subgroup relations, can only be applied to transitions where no long range diffusion occurs, i.e., when a single phase transforms to a single phase of the same composition. For equilibrium transformation in multi-component systems, this can only occur at special compositions (consolute points) or for second or higher order transitions [2]. However, partitionless transformations can also occur during cooling for first order transitions (for alloys with equilibrium multiphase fields) when sufficient undercooling of the high temperature phase is achieved that a limit of metastability is reached. This limit defines a temperature where the metastable undercooled phase becomes unstable and spontaneous transformation occurs with no long-range diffusion requirement (congruent ordering) [3–5]. Experimentally, such congruent ordering could occur during continuous cooling if the cooling rate is fast enough to prevent competing transformations involving long-range diffusion. Clearly a mechanism for fast transformation kinetics must be available for these partitionless transformations, i.e., fast short-range diffusion in chemical (substitutional or interstitial) ordering or mobile defect motion producing the necessary atomic displacements in displacive (martensitic) ordering.

In this paper, experimentally observed microstructures of three alloys from and near the pseudo-binary (Ti,Nb)_3_Al section will be analyzed. The alloys are Ti-25Al-12.5Nb (at%) (Alloy 1), Ti-25Al-25Nb (at%) (Alloy 2) and Ti-28Al–22Nb (at%) (Alloy 3). The alloys are close in composition to some alloys studied by different research groups in attempts to develop titanium aluminides for aerospace high temperature structural applications (see Refs. [1–22] in [1]). We believe that the approach used here where the transitions are viewed as a sequence of symmetry changes will provide a unified view of the complex microstructural transitions in these materials. The microstructures studied, as represented by the distribution, type and morphology of interfaces induced by the transformations, will be compared with the interfaces predicted by the theoretical considerations of [1]. The predictions represent idealized transformations, without taking into account such complications as the temporary coexistence of parent and transformed phases, or the mobility of the interfaces created. Therefore the predictions will be considered primarily as a point of reference for comparison to the details of the actual transformation process. The main goal of the paper is to demonstrate that the microstructures of the alloys studied correspond very closely to two of the possible transformation paths predicted:

For Alloy 1–with intermediate hexagonal phases, [ Im3¯m(A2) →Cmcm(A20)→P6_3_/mmc(A3) →P6_3_/mmc(DO_19_)→Cmcm(O)],
For Alloys 2 & 3–with possible intermediate orthorhombic B19 structure [ $\mathrm{Pm}\bar{3}m(B2)$→Pmma(B19)→Cmcm(O)].

In addition to the study of the diffusionless transformations, the effect of prolonged annealing, which results in a compositional phase separation, has been also investigated for the Ti-25Al-12.5Nb alloy. In this case the mechanism of microstructure formation becomes clear when the thermodynamic principles of both ordering transformations and phase separation in systems where both order and composition parameters variables are used [3,5].

## 2. Experimental

### 2.1 Specimen Preparation

Three alloys with the compositions Ti-25A1-12.5Nb (at%) (Alloy 1), Ti-25Al-25Nb (at%) (Alloy 2) and Ti-28Al-22Nb (at%) (Alloy 3) were prepared by arc melting. A minimum of ten remelts was necessary to ensure mixing of the components. All samples received a homogenization treatment at 1400 °C for 3 h in a vacuum tight furnace under 2/3 atm of gettered Ar. During heat treatment samples rested on a Y_2_O_3_-coated Al_2_O_3_ substrate supported on a moveable pedestal which could be lowered out of the hot zone of the furnace into a lower chamber. The cooling rate of the samples during such cooling was estimated to be about 400 °C/min. SEM microprobe of these samples using elemental standards gave the following compositions: (Alloy 1) Ti-24.7Al-12.6Nb (at%) (Alloy 2) Ti-23.2Al-25.8Nb (at%); and (Alloy 3) Ti-27.9A1-22.8Nb (at%). Typical oxygen, nitrogen and hydrogen levels for this procedure were less then 500, 350, 40 wppm, respectively.

For the study of the partitionless transformations, samples were examined after additional annealing at 1100 °C for 4 d and cooling to room temperature at two rates: at about 400°C/min in the furnace described above or by water quenching. These latter samples were heat treated in another furnace in evacuated and He-backfilled quartz tubes after being wrapped in Ta foil. As the results will show, the 400 °C/min cooling rate was slow enough to permit complete transformation to the orthorhombic phase for Alloy 1. However only partial transformation occurs for Alloys 2 and 3. Therefore samples of these two alloys were given a subsequent annealing at 700 °C for 15 min in quartz tubes. To determine the phase equilibrium at 700 °C, the samples annealed at 1100 °C were additionally annealed at 700 °C for different lengths of time, up to 26 d in quartz tubes.

TEM foils were prepared by a standard twin-jet electropolishing procedure using a 300 ml methanol, 175 ml *n*-butanol and 30 ml HClO_4_ electrolyte at 0 °C. Optical metallography was performed by mechanical polishing and subsequent etching with Kroll’s reagent.

### 2.2 The Problem of Artifact Structures in Ti Alloy Thin Foil (TEM) Specimens

The formation of artifact structures in different Ti alloys during electropolishing of TEM specimen has been discussed in the literature [6–13]. Charging of thin foils by hydrogen in the course of electrochemical thinning was found to be possible. The concentration of hydrogen may be sufficient to cause formation of either different hydrides and/or hydrogen-stabilized martensites. Artifact structures such as fcc, fct, hcp and orthorhombically distorted *α*_2_ have been reported. The amount of the accumulated hydrogen depends on a specimen’s thickness prior to electropolishing, and on the type and temperature of the electrolyte. It was claimed [7] that the charged hydrogen may escape from a thin foil after electropolishing, unless the foil is protected by an oxide layer. If hydrogen escapes the specimen, the reversion of the hydride may result in the formation of 1/2〈111〉 dislocation loops in the BCC phase.

In order to be confident that microstructures observed by TEM in this study do not contain the described artifacts, several TEM specimens of the same material were prepared for comparison by two additional thinning techniques, presumably not affected by hydrogen contamination. The first technique was twin-jet electropolishing with non-acid electrolyte containing a solution of CaCl_2_ in methanol [14]. The second technique was mechanical grinding to a 30 µm thickness with a dimpler followed by ion-milling. Specimens prepared by these two techniques show microstructures similar to those of the specimens prepared by an acid-based electrolyte.

## 3. High-Temperature Phases: Microstructure After Water Quenching From 1100 °C

The identity of the high temperature phase was evident from the microstructural examination of specimens water quenched from 1100 °C. Optical examination revealed large equiaxed grains that appeared a single phase. According to selected area electron diffraction (SAD) at room temperature, the phase has B2 order for all three alloys.

For Alloy 1 the presence of a high density of anti-phase boundaries (APBs) (observed using a dark-field image with a superlattice 100 reflection) suggests that at 1100 °C the high temperature phase was disordered BCC. The cooling rate during quenching was fast enough to prevent formation of the low temperature phases but not the ordering and coarsening of the anti-phase domains (APDs). Typical of B2 ordering, the APBs have a two-domain interconnected morphology with isotropically curved interfaces. From these studies it is not clear whether the BCC to B2 transition in the Ti-Al-Nb system is first order (with a BCC + B2 two phase field) or second order.

For Alloys 2 and 3 no APBs due to the BCC→B2 ordering were observed after quenching. This fact suggests that the B2 order for these compositions exists at 1100 °C (in fact up to 1400 °C [15]).

We have referred here to the quenched-in phase as being cubic B2. However this is strictly correct only if local displacements of atoms from positions of cubic symmetry are ignored. The effect of such displacements are readily observed as an overall “tweed” contrast in TEM images for all three alloys (This tweed is known in the literature for different alloy systems as pre-martensitic, or pre-transformation phenomena [16]). Due to these displacements, the SAD patterns from all three alloys contain diffuse scattering: distortion of the cubic reflections, streaking along 〈011〉* and $\langle 1\bar{1}2\rangle *$ directions, and loci of diffuse intensity close to 1/2〈011〉* and $1/2\langle 1\bar{1}2\rangle *$ positions in reciprocal space. As will be seen later, these are the positions where reflections from different crystallographic variants of the O phase will occur. For Alloy 3 additional weak diffuse scattering near 1/2〈111〉* positions is probably due to ω-type distortions [17,18]. In addition to the “tweed”, defects similar in contast to dislocation loops are seen occasionally for all three alloys and are believed to be related to the nucleation mechanism of the low temperature phases.

## 4. Microstructures Due to the Diffusionless Transformation of the High Temperature Cubic Phase to the Orthorhombic O Phase

### 4.1 Microstructure of Alloys 2 and 3 Corresponding to the Transformation Path $\mathrm{Pm}\bar{3}m(B2)$→Pmma(B19)→Cmcm(O)

From the transformation paths suggested by the symmetry considerations in [1], the Im3¯m(A2)→ $\mathrm{Pm}\bar{3}m(B2)$→Pmma(B19)→Cmcm(O) path (2) is the only one expected when B2 ordering precedes the transition to the close-packed structure. Here we will demonstrate that the experimental evidence from TEM supports this formal supposition for both Alloys 2 and 3 which have the B2 structure as the high-temperature parent phase. The observed distribution and type of interfaces of the O phase correspond to those shown schematically in Fig. 9 of Ref. [1], with the exception that this schematic presumed the parent phase was BCC and thus includes the APBs due to the BCC→B2 ordering. Microstructures similar to those observed here, but with B2 APBs, have been observed recently for a Ti-24Al-15Nb (at%) alloy where the parent phase was indeed disordered BCC (Ref. 8 from[1]).

#### 4.1.1 Formation of the Plate-Like Domains of the O Phase

**Fig. 1:**
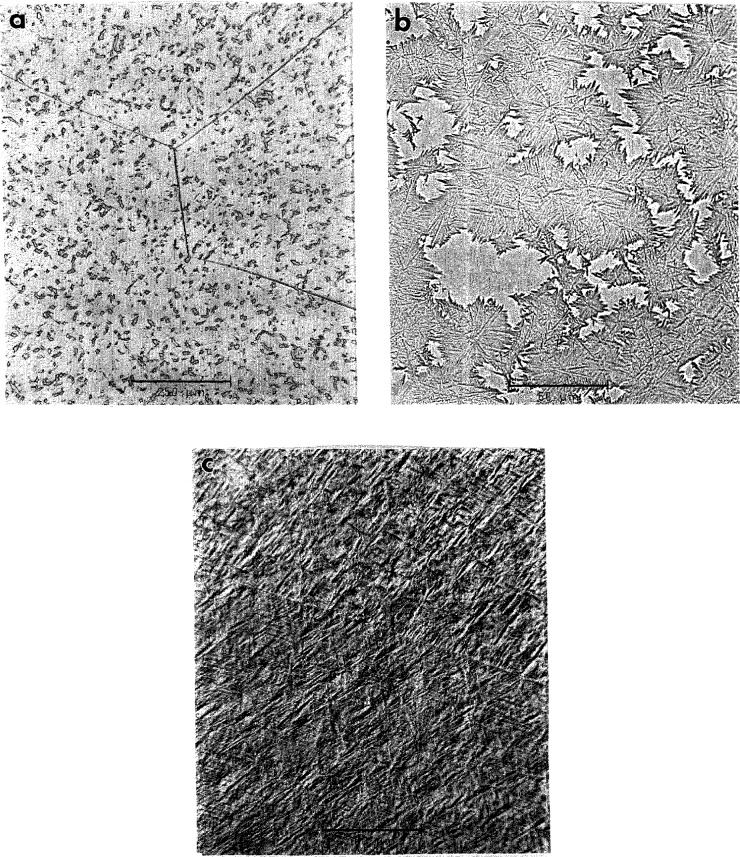
Optical micrographs of Alloy 3 specimens cooled from 1100 °C at different cooling rates. Regions with different volume fractions transformed according to (a) a higher and (b) a slower cooling rate, (e) Annealing of the water quenched specimens (with retained B2) for 15 min at 700 °C was sufficient to produce complete transformation.

For Alloys 2 and 3 the kinetics of transformation of the B2 phase to a low temperature phase was found to be relatively sluggish as is evident from optical micrographs, Fig. 1a, b. The micrographs show regions of partially transformed material, differing in their volume fraction according to the differences in cooling rates of the specimens. Annealing of the water quenched specimens (with 100% retained B2 phase) at 700 °C for 15 min was sufficient to produce complete transformation (Fig. 1c). Apparently the transformation proceeds by copious nucleation where the transformed regions grow uniformly outward until impingement. The transformation is partitionless without measurable difference in composition between the parent and transformed regions.

**Fig. 2:**
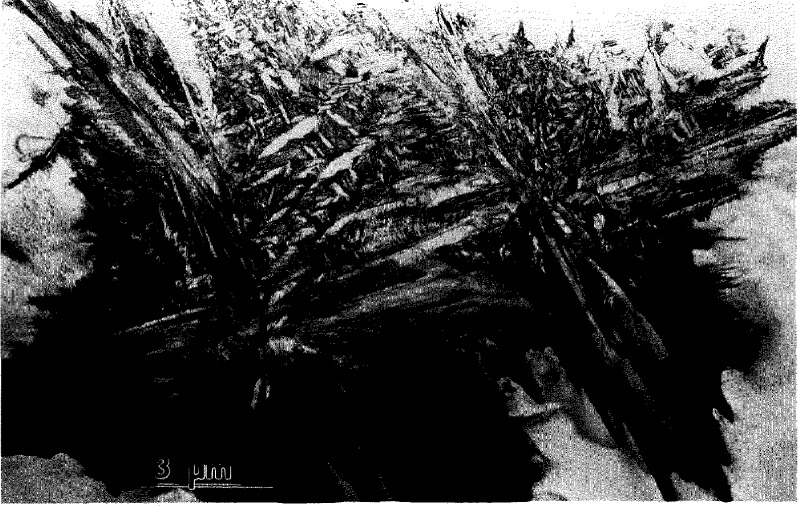
The TEM microstrueture of the Alloy 3 specimen corresponding to Fig. 1a. The TEM image shows islands of transformed material, surrounded by a B2 phase matrix. The islands consist of a complex plate-like structure of the O phase. The phases do not differ in composition.

**Fig. 3:**
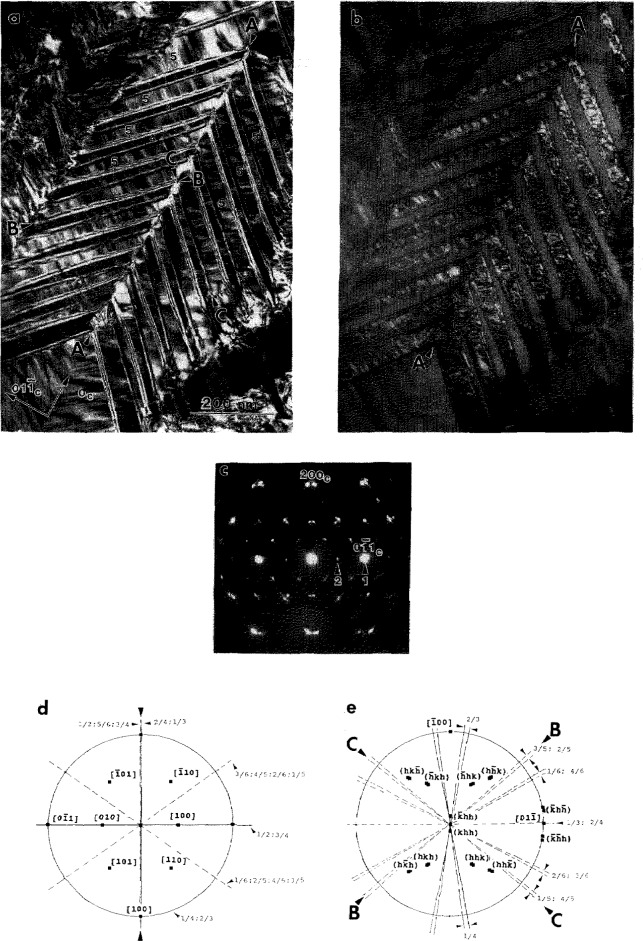
Higher magnification view of the plate-like structure of Fig. 2 showing the plates to be arranged into a larger size secondary plate (polytwin) which alternates with another variant of polytwin plate, (a) and (b) are dark-field images with the g_1_ and g_2_ reflections, indicated in the (c) [011]_c_ SAD pattern. The gz reflection, 020_o_, images a single variant (5) of the O phase. (d,e) show [011]_c_ stercographic projections with superimposed calculated [1] traces of the (d) symmetric and (e) non-symmetric SFIs and the corresponding traces of the observed A-A, B-B and C-C interfaces of (a). The solid and dashed traces correspond to edge-on and inclined interfaces respectively.

From TEM observations of the partially transformed specimens it is evident that the transformed regions have a complex microstructure of plate-like domains (Fig. 2). The smallest plates typically form an alternating sequence packed in a region named a polytwin in Ref. [1]. The polytwins themselves often have a plate-like shape and alternate with similar polytwin plates, as shown in Fig. 3a, b. Growth of the plate-like structure into the B2 phase matrix appears to have a common but ragged and diffuse transformation front (Fig. 2). Only occasionally were independently grown single-domain plates observed. Therefore in most cases the growth of a plate is not independent but is correlated with the formation and growth of neighboring plates having rotational variants able to accommodate transformation strains.

**Fig. 4:**
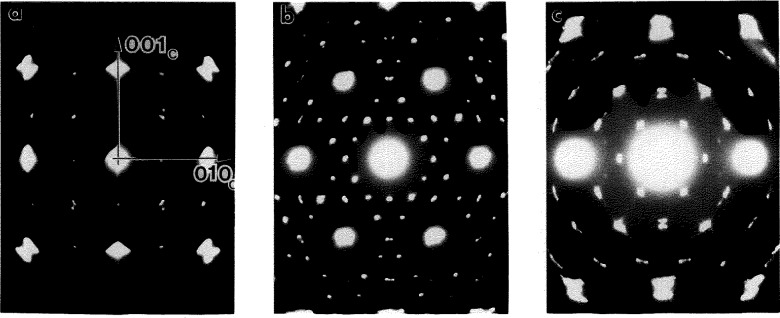
A series of SAD patterns taken from an Alloy 2 specimen which was water quenched from 1100 °C and then annealed at 700 °C for 15 min. The selected area aperture was large enough to include numerous variants eontrihuting to scattering. The patterns show average Laue symmetries (a) 4 mm, (b) 3 mm and (e) 2 mm corresponding to the major zone axes of the cubic symmetry, [100], [111] and [110], respectively. The strongest reflections (consisting of several reflections from different variants of the O-phase) correspond to the fundamental BCC reflections and determine the orientation relationship hetween lattices of the transformed B2 phase and the O phase variants.

Selected area electron diffraction (SAD) (Figs. 3c and 4) combined with convergent beam (CB) electron diffraction and powder neutron diffraction [19] confirm the plates to be the O phase. No other phases were found in the samples that were continuously cooled or in those annealed at 700 °C. From the SAD patterns of Fig. 4 a lattice correspondence between the B2 and the O phase is evident as the common one for BCC and close-packed structures [20]:

$$\begin{array}{c} {\left[001\right]}_{o}∥{\left[011\right]}_{c}\text{and}{\left[100\right]}_{o}∥{\left[100\right]}_{c}\\ (c–\text{cubic};\;o–\text{orthorhombic}). \end{array}$$

The correspondence is the same as that used for the subgroup scheme of Ref. [1]. It gives six rotational variants of the orthorhombic phase (either B19 or O for path 2), each with its basal (001)_o_ plane parallel to one of the six {110}_c_ planes of the parent cubic structure. Small mutual rotations of the contacting variants are necessary to accommodate the transformation strains (self-strains) by creating stress-free interfaces (SFIs), as discussed in [1].

The microstructure of sufficiently large volume has an average cubic symmetry due to the presence of all six rotational variants of the O phase. The symmetry is clearly seen in the SAD patterns of Fig. 4a, b, c showing (a) 4 mm, (b) 3 mm and (c) 2 mm average Laue symmetries corresponding to the major zone axes of the cubic symmetry, [100]_c_, [111]_c_ and [110]_c_. These average axes indicate the orientation of the parent (transformed) B2 phase lattice.

#### 4.1.2 Stress Accommodating Morphology of the O Phase

All interfaces between the pairs of variants in and between the polytwin plates are expected to be SFIs, as discussed in [1]. The pair of polytwins shown in Fig. 3 will be analyzed in order to demonstrate that the interfaces are indeed described as SFI. The analysis will be performed in coordinates of the parent cubic lattice. Two polytwin plates are seen in Fig. 3a, with the planar A-A interface between them [for a [011]_c_ orientation of the thin foil, (Fig. 3c)]. The A-A interface has (011)c orientation and is “edge-on.” The individual plates in the polytwins have nearly parallel inclined interfaces (B-B and C-C sets) between the variants. The interface traces are approximately ±45° to the [100]c direction. The plates in each polytwin are nearly mirror related across the ${\left(01\bar{1}\right)}_{c}$ plane, and therefore there is an apparent continuity of the plates across the A-A polytwin interface.

Dark field imaging with the 020o reflection (Fig. 3b) proves that the plates labeled 5 and 5′ in Fig. 3a from each polytwin belong to the same variant 5 (the variant labeling follows the scheme described in [1]). The variant is oriented with [001]_o_ parallel to [011]_c_ (the beam direction) and the 020_o_, 200_o_ and 110_o_ reflections of the variant do not overlap with reflections from the other variants. Misorientation between the 5 and 5′ plates (around a common [001]_o_) is measured as about 10° (Fig. 3c). According to microdiffraction, the remaining two plate orientations are close to (212)_o_ and belong to any pair chosen from among the 1, 2, 3, or 4 (not 6) variants [1].

If the structure shown in Fig. 3 is coherent and strain accommodating, the observed interfaces are expected to correspond to the SFIs calculated in [1]. Referring to Fig. A.3 in Appendix B of [1] (reproduced here as Fig. 3d,e) where the traces of the SFIs for the [011]_c_ zone axis are given, we conclude (according to the measured angle of the trace and the widths of the B-B and C-C interface projections) that the B-B interface corresponds to $hh\bar{k}\;(\text{or}\;hk\bar{h})$ between variants 3/5 (or 2/5) and the C-C $h\bar{k}h$ $\left(\text{or}\;h\bar{h}k\right)$ interface corresponds to between variants 1/5 (or 4/5) (Fig. 3d, e). The ambiguity of choice between the pair of variants in the polytwin plate can be resolved if one determines what side of the projected B-B and C-C interfaces intersect the upper and lower surfaces of the TEM foil. In order to have the line of intersection of the B-B and C-C planes lie within the A-A plane (as Fig. 3 suggests), the combination of variants must be either 4/5 and 2/5 or 1/5 and 3/5. These interfaces are irrational (twins of the II kind) and therefore their exact orientation depends on the lattice parameter of the orthorhombic phase at the temperature of transformation (Fig. 3e shows the spread of possible orientations for lattice parameters varying between Ti_2_AlNb and DO_19_ phases, see Ref. [1]).

The A-A interface consists of alternating segments that are structurally different. One type of segment is a ${\left(01\bar{1}\right)}_{c}$ interface between 4 (or 1) and 2 (or 3) variants. This is a symmetric SFI (twin of the I kind). The second type of segment separating variant 5 (5′) misoriented plates is a low angle symmetric boundary (~ 10°) which may be relaxed by forming a dislocation wall.

All high angle interfaces in the polytwin microstructure in Fig. 3 are thus shown to be SFIs. Observation of such an elastic energy accommodated arrangement of plates suggests an interacting process during their formation, i.e., a process where the presence of certain combination of variants in one polytwin plate influences the formation of variants in a neighboring plate. Formation of a strain accommodating arrangement of twins, of both the I and II kind, is an important phenomena in martensitic transformations of numerous shape-memory alloys [21–23].

#### 4.1.3 Substructure of the O Phase Primary Plates

**Fig. 5:**
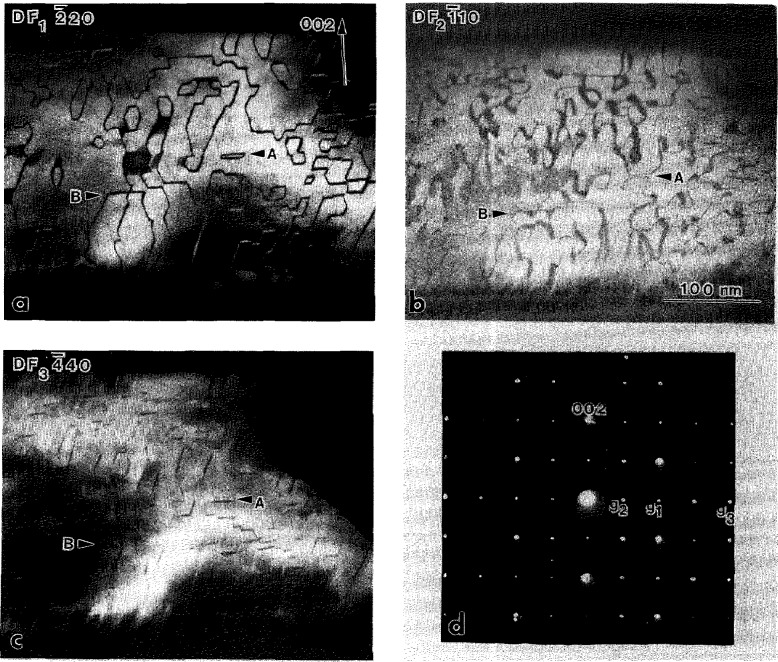
Three dark field (a–c) images taken from a single variant plate in specimen of Alloy 3 continuously cooled from 1100 °C. The dark field images are taken with the plate orientation close to the (d) [110]_o_ zone axis using (a) $\bar{2}20_{o}$, (b) $1\bar{1}0_{o}$ and (d) $\bar{4}40_{o}$ reflections in approximately a two-beam condition. Two type of interfaces with different displacement vectors and morphology are observed. Both types arc seen in (b) and only the faceted type in (a). In (c) only residual contrast from the faceted interfaces is seen, Arrow markers A and B identify the same places in all three micrographs.

Closer examination of Fig. 3a, b reveals a substructure of a relatively high density of interfaces (defects) inside the plates of different variants of the O phase. The interfaces are of a translational type since they do not affect the positions of the reflections in each single variant. Dark field imaging was used to determine the ***R*** value using standard ***R***·***g*** *=n* (n=0,±l,±2..) invisibility criteria (***R*** is the translation vector between two domains separated by the interface, ***g*** is an operating dark field reflection). Figure 5a, b, c shows three dark field images taken from a single variant oriented close to the [110]_o_ zone axis (SAD pattern in Fig. 5d). Two types of interfaces, different in their displacement vector ***R*** and morphology, are seen in these images. The presence of these interfaces are in accord with the transformation path 1 in [1].

The interfaces of the first type, with a wavy APB appearance, are visible with the superlattice reflections of the O phase, e.g., $\bar{1}10_{o}$ in Fig. 5b, but are invisible with the fundamentals, e.g., $\bar{2}20_{o}$ and $\bar{4}40_{o}$ in Fig. 5a,c. The second type has a distinct faceted appearance (clearly seen in Fig. 5a) and is visible with both the superlattice and some fundamental reflections, e.g., $\bar{1}10_{o}$ and $\bar{2}20_{o}$ (Fig. 5a,b). Both types are invisible with 002_o_. For the $\bar{4}40_{o}$ reflection the faceted interfaces has only residual contrast (Fig. 5c). The experimental results on the visibility for both interfaces are summarized and analyzed for various displacement vectors in Table 1.

Analysis of the ***R***·***g*** product for the first type suggests that the displacement vector ***R*** is l/2[010]_o_. (C-centering of the Cmcm space group of the O phase makes the l/2[100]o vector an equivalent one.) This displacement vector is close to the antiphase vector between two differently ordered 4c_1_ and 4c_2_ sites in the pseudo-hexagonal (001)_o_ plane of the O phase. Such ABPs may result after ordering of the B19 orthorhombic structure to the O phase structure (leading to a doubling of the ***a***, ***b*** unit cell parameters) and are expected in the last step of the transformation path 2. The ordering requires a separation of the mixed (Al,Nb) site of the B19 (and B2) structure into predominantly Al (4c_1_) and Nb (4c_2_) sites in the O phase (see Fig. 3 of [1]). Such ordering will produce a two domain interconnected morphology with no triple junctions as schematically shown in Fig. 9 of [1]. The morphology is similar to that seen in Fig. 5b, where the curved APB interfaces appear as either closed loops (with enclosed volume) or are attached to other interfaces. The presence of the l/2[010]_o_ APBs provides the only evidence for the existence of the possible transient B19 structure.

The invisibility of the second type of interfaces with the 002 reflection suggests that their displacement vector, ***R****_2_*, has a *z*-component that is either 0 or 1/2. Other visibility conditions (Table 1) correspond to the [0 1/4 l/2]_o_ (or [0 1/4 0]_o_) displacement vector of a two-domain structure. Indeed, as Fig. 5a shows, no triple junctions of the faceted interfaces are seen, and the interfaces form either interconnected or closed volumes. The [0 1/4 l/2]_o_ vector in the smaller unit cell of the orthorhombic Cmcm(A20) or Pmma(B19) structures (with ***a*** and ***b*** parameters half of the O phase) corresponds to an unique vector [1/2 0 l/2]_B19_. As it has been discussed in [1], such displacement between domains is expected for the B2 to B19 transition, and therefore the faceted interfaces are due to the first step of the transformation path 2.

The B2 to B19 (or similarly BCC to A20) transition is of a displacive type, with both homogeneous and heterogeneous (shuffle) transformation strain components [1]. The homogeneous strain determines the orientation of the six twin variants of the orthorhombic or hexagonal structure, as was discussed in Sec. 4.1.1. The translational [1/2 0 1/2]_B19_ domains result from shuffles acting in opposite directions ([24], Ref. [8] in [1]). An interface between such translational domains is expected to have structural relaxation toward the close-packed stacking faults. This is supported by the observation of residual contrast for the 440_o_ reflection (Fig. 5c) suggesting small displacements in addition to [0 1/4 l/2]_o_. The faceted interfaces were analyzed by trace analysis in order to find their orientations. Two different crystallographic planes for the facets were found: the basal (001)_o_ and close to the {221}_o_.^1^

**Fig. 6:**
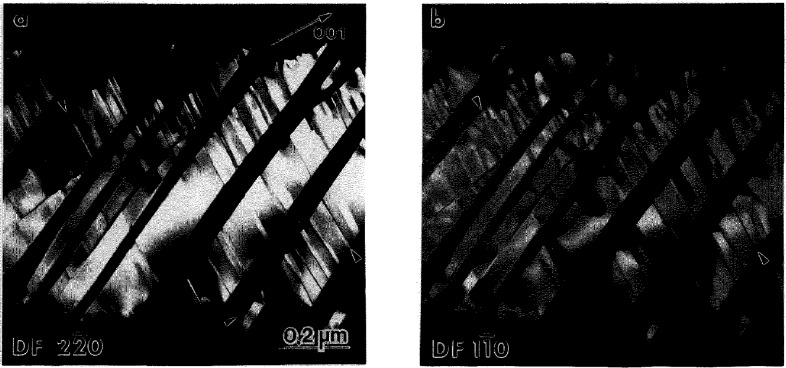
Dark field images of (a) stacking fault (SF) type and (b) both SF and APB interfaces in thin plates of an O phase variant oriented with [110]_o_ parallel to the electron beam. In (a) and (b) $2\bar{2}0_{o}$ and $1\bar{1}0_{o}$ reflections were used respectively.

When the twin plates are thin, the faceted interfaces appear as a sequence of stacking fault planes rather than domain boundaries. Dark field imaging of the interfaces (Fig. 6a) shows that they are (001)_o_ planes. The APBs are seen as stretched between the twin boundaries (Fig. 6b), probably because of surface energy considerations (balance of interfacial energies of APB and twin-type interfaces at a triple-junction).

### 4.2 Microstructure of Alloy 1 Corresponding to the Transformation Path Im3¯m(BCC) → Cmcm(A20)→P6_3_/mmc(A3)→P6_3_mmc(DO_19_) →Cmcm(O Phase)

Alloys with compositions close to Alloy 1 exist at high temperature as a disordered BCC phase [15,25]. According to the theoretical considerations of [1], if the BCC does not order to B2 prior to the displacive transition to a close-packed structure, two transformation paths, 1 and 3, are possible. Here we will show evidence supporting the transformation path 1 for Alloy 1:

Im3¯m(BCC)→Cmcm(A20)→P63/mmc(A3)→P63mmc(DO19)→Cmcm(O phase).

The main feature of the path is the formation of intermediate hexagonal symmetry phases. This causes the O-phase rotational variants to be related to each other not only by the cubic symmetry of the parent phase but also by hexagonal symmetry.

#### 4.2.1 Transformation to the Coarse Needle-Like Structure During Continuous Cooling

**Fig. 7:**
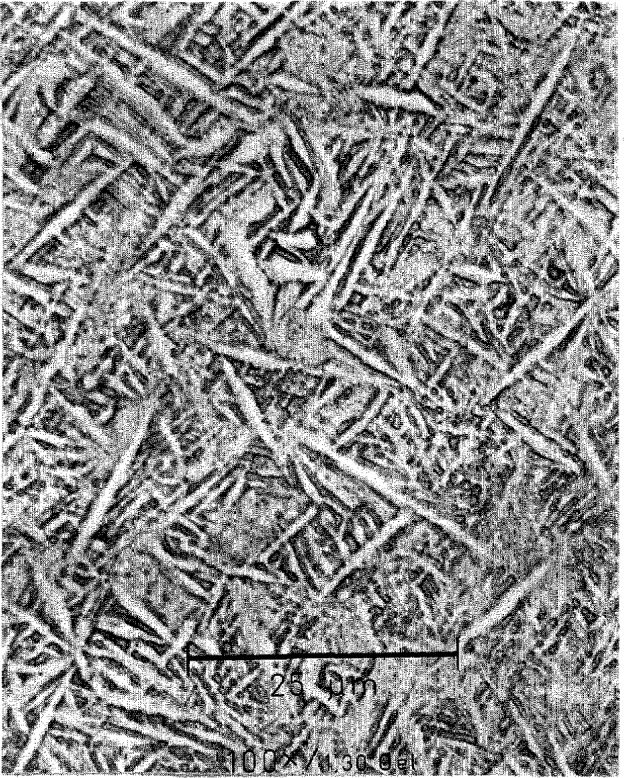
Optical micrograph showing a needlc-like transformed microstructure of Alloy 1 continuously cooled from 1100 °C at 400 °C/min.

**Fig. 8:**
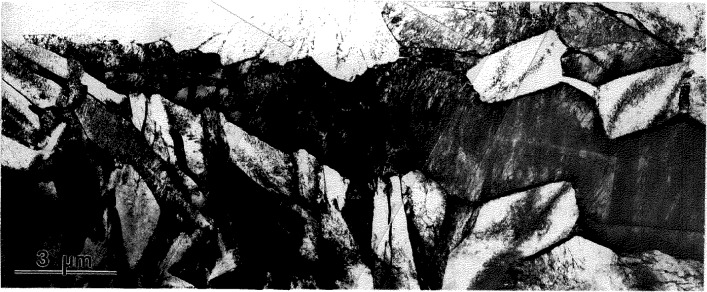
Bright field TEM micrograph showing the detailed morphology of the needles shown in Fig. 8. The needles have very irregular interfaces. Blocky grains between elongated needles are most probably cross-sections of the needles with their long axes normal to the TEM foil.

**Fig. 9:**
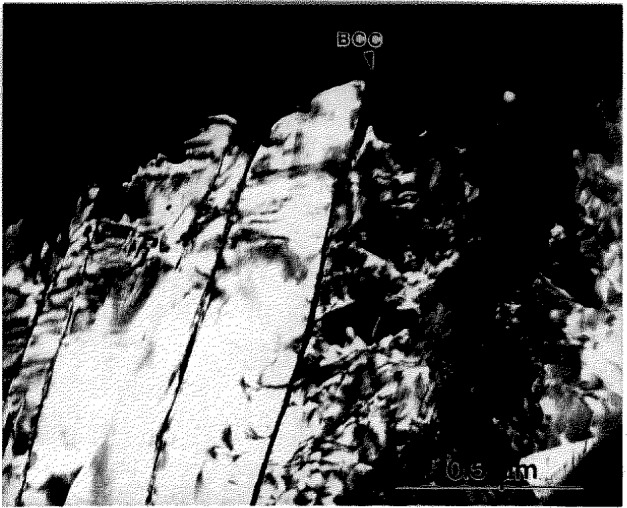
Thin (dark) layers of the BCC phase separating transformed phase grains. The dark-field image is taken with a DO_19_ reflection which is well separated from the BCC’s. Dark contrast in the upper-left part of the photograph belongs to a different grain.

For Alloy 1 the transformation kinetics are significantly faster than are those for Alloys 2 and 3, and therefore cooling from 1100 °C to room temperature at a rate of 400 °C/min was slow enough to complete the transformation of the high temperature phase to the close-packed structure. The transformed microstructure has a morphology resembling a martensite structure when observed by optical metallography (Fig. 7). The TEM micrograph in Fig. 8 shows in more detail that the morphology is rather needle-like. The needles have very irregular interfaces (not resolved in Fig. 7), and no crystallographic habit plane. This is in great contrast to the mostly regular plate-like structure seen in Alloys 2 and 3. The blocky grains surrounding an elongated needle in Fig. 8 are probably cross-sections of needles oriented with their long axes parallel to the electron beam direction. The wavy character of the interfaces seems to be the result of impingement of needles during their independent growth. In a few regions, grains were found to be separated from each other by a thin layer identified as the BCC phase (Fig. 9). Enrichment of Nb in the BCC layer was detected by EDS for this and similar alloys [25]. The observed morphology suggests that near impingement, some diffusion does occur during the growth of the needles. We presume that in these regions the competing transformation with long range diffusion is marginally possible. Thus the cooling rate of these samples is on the lower limit for the dominance of the partitionless transformation.

**Fig. 10:**
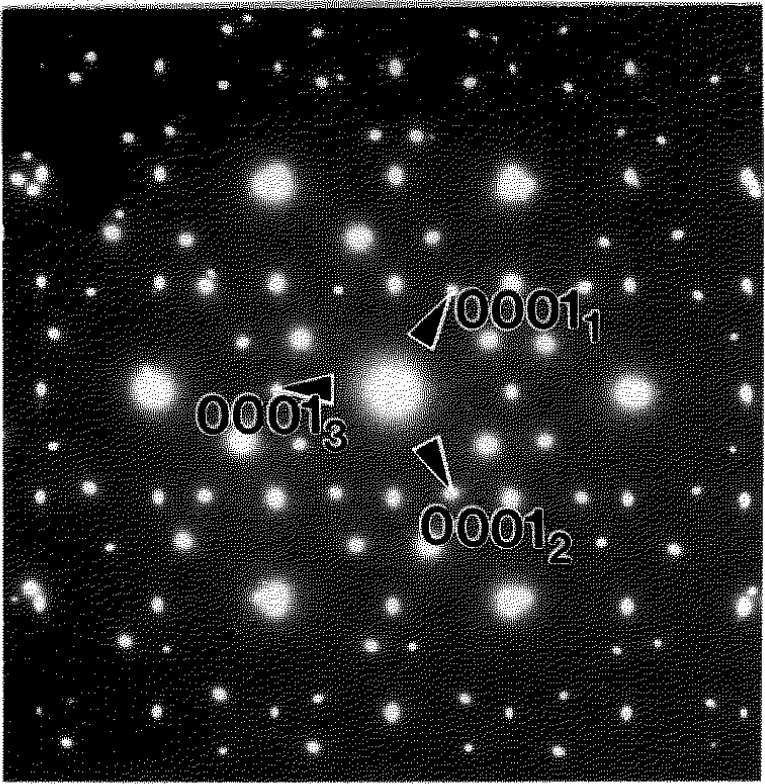
SAD pattern taken from three grains forming a triple junction in the Alloy 1 cooled from 1100°C. The pattern has ${\left[10\bar{1}0\right]}_{h}∥{\left[111\right]}_{c}$ (three 60° rotated ${\langle 10\bar{1}0\rangle }_{h}$ patterns around [111]_c_) and (0001)_h_||{110}_c_ in support of the Burgers OR found for the Alloys 2 and 3.

Selected area diffraction from individual needles correspond to the reciprocal lattice of the DO_19_ ordered hexagonal structure. The orientation relationship between different variants of the hexagonal structure and the previously existing BCC phase can be inferred from Fig. 10, which is taken from three neighboring grains forming a triple junction. The present pattern is indexed as three variants of the DO_19_ phase (h) with ${\left[10\bar{1}0\right]}_{h}∥{\left[111\right]}_{c}$ and (0001)_h_||(110)_c_. This is the known Burgers orientation relationship [21].

Close examination of the SAD patterns reveals splitting of the spots and diffuse streaking in and normal to the (0001)_h_ plane. These diffraction effects come from the substructure inside the needles, which is seen as dark contrast in Fig. 8. The nature of the spot splitting indicates the existence of orthorhombic distortions in the basal plane of the DO_19_ phase and suggests the presence of O phase domains. Therefore the hexagonal indexing that we use describes only the average symmetry and orientation of the needles.

The needle-like structure is not a stress accommodating plate-like structure as observed in Alloys 2 and 3. This difference may perhaps be understood by considering the possibility that the transformation of the BCC phase of Alloy 1 may occur at higher temperature than Alloys 2 and 3 as indicated by the occurrence of some small level of long-range diffusion near impingement. At higher temperatures the ductility of the phases may be sufficient to accommodate the transformation stresses by plastic deformation (including formation of the observed stacking faults) and negate the requirement to form a stress accommodating structure. Indeed a dependence of morphology on cooling rate has been observed for alloys similar in composition to Alloy 1 [25] that range from those accommodating stress at high cooling rates to those similar to the morphologies described here at lower cooling rates. The occurrence of similar orientation relationships in both diffusionless and diffusion controlled (precipitation) transformations is well known and is in fact observed in some other Ti-Al-Nb alloys [15].

#### 4.2.2 Substructure of the Needles

**Fig. 11:**
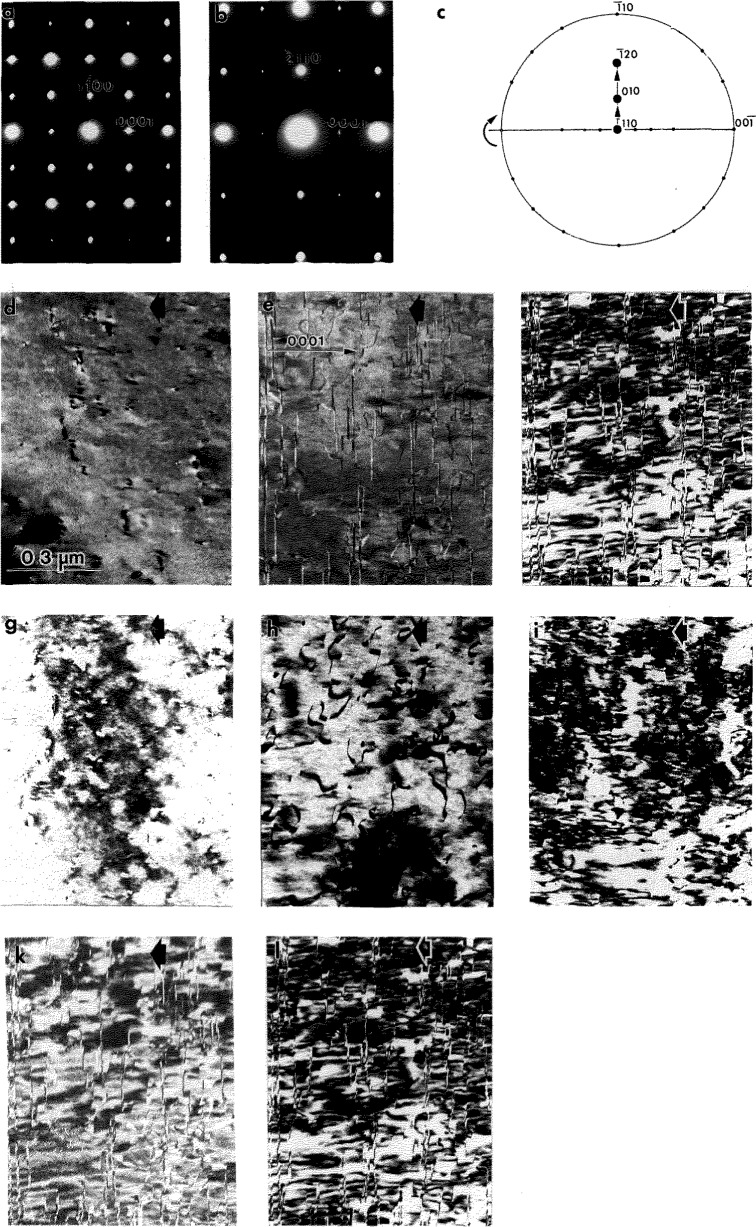
Series of dark field images taken from a single grain of the transformed Alloy 1 in the ${\left[11\bar{2}0\right]}_{h}$, ${\left[10\bar{1}0\right]}_{h}$ and ${\left[\bar{1}210\right]}_{h}$ zone axis orientations (SAD patterns of ${\left[11\bar{2}0\right]}_{h}$, and ${\left[01\bar{1}0\right]}_{h}$ patterns are shown in (a) and (b)). The sequence of orientations was obtained by tilting the grain around the [0001]_h_ direction as shown in the stereographie projection (c). The dark field images are taken close to a two-beam condition using the following reflections: (d) 0002, (e) $1\bar{1}00$ and (f) $2\bar{2}00$ from the ${\left[11\bar{2}0\right]}_{h}$ (g) 0002, (h) $2\bar{11}0$ and (i) $4\bar{22}0$ from the ${\left[01\bar{1}0\right]}_{h}$ ZA; (k) $10\bar{1}0$ and (1) $20\bar{2}0$ from the ${\left[\bar{1}2\bar{1}0\right]}_{h}$ ZA.

Similar to the result found for Alloys 2 and 3, the substructure of the needles has two types of interfaces associated with the translational domains. The interfaces have been imaged in dark field with different reflections belonging to three zone axes, ${\left[11\bar{2}0\right]}_{h}$, ${\left[01\bar{1}0\right]}_{h}$ and ${\left[\bar{1}210\right]}_{h}$, by tilting a single grain around the [0001]_h_ direction, starting from the ${\left[11\bar{2}0\right]}_{h}$ zone axis (Fig. 11). As with Alloys 2 and 3, two types of interfaces are morphologically distinct: one has curved isotropic APB-type interfaces (Fig. 11h) and the other has planar interfaces. However the planar interfaces only occur in (0001)h planes for Alloy 1 (Fig. 11f) as compared to the closed (or interconnected) surfaces observed for the Alloys 2 and 3. The observed visibility conditions for both types of interface are similar to those reported in [8] (see Ref. [1]).

The wavy isotropic interfaces are APBs between the domains formed by ordering of the disordered hexagonal structure to the DO_19_ structure [26]. The APBs have $R=\left(1/6\right){\langle 11\bar{2}0\rangle }_{h}$ displacement vectors. For the three such ***R*** vectors equivalent under the 6-fold symmetry operation, there are three different APBs and four distinct translational domains. (Compare this to the single APB and two domains observed in Alloys 2 and 3). When the APBs are imaged with superlattice reflections, only two of the three APBs are visible according to the ***R***·***g*** conditions. Therefore no triple junctions of the APBs can be seen. Comparison of the two dark field micrographs shown in Figs. 11e and Figs. 11he, show that some APB segments are visible for both of these imaging conditions $\left(\text{for}\;R=1/6{\left[\bar{1}2\bar{1}0\right]}_{h}\right)$, while some segments are visible for only one of the imaging conditions. Therefore, the presence of the disordered hexagonal A3 as an intermediate state is established by the presence of the $1/6{\langle 11\bar{2}0\rangle }_{h}$ APBs, which can only be due to the P63/mmc(A3)→ P6_3_mmc(DO_19_) transition.

The second type of interface, with a planar morphology, was identified as having $1/6\langle 20\bar{2}3\rangle$ displacement vector. These interfaces give rise to the [0001]* streaking. Because the interfaces terminate inside the needles, they are rather stacking fault defects than boundaries of translational domains (like in the Alloys 2 and 3). The stacking faults may provide an inhomogeneous shear necessary to satisfy conditions of the invariant plane strain [21,22].

#### 4.2.3 Congruent Ordering of the DO_19_ to O Phase

**Fig. 12:**
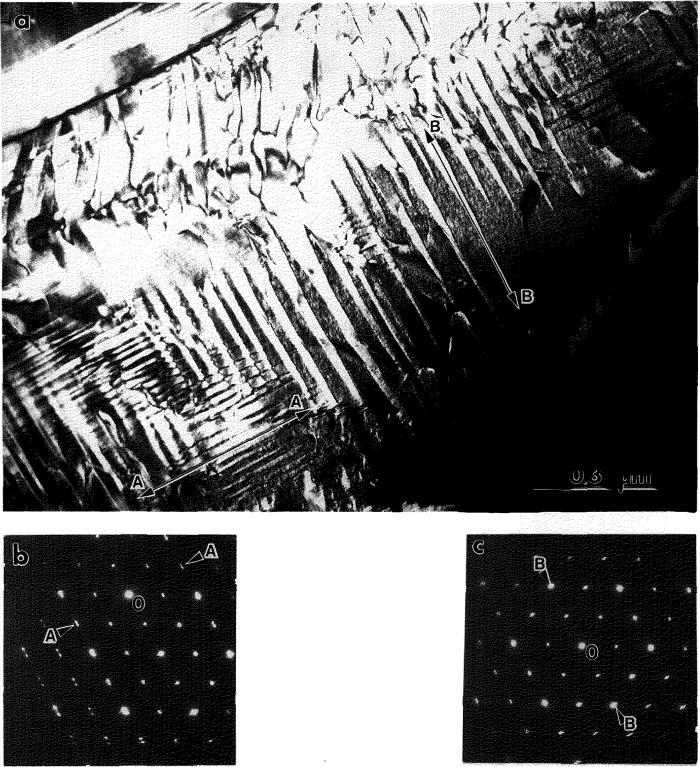
(a) TEM dark field image of a continuously cooled Alloy 1 specimen, slightly off the [0001]_h_ zonc axis in order to give different excitation errors for different domains. Two orthogonal directions of the interface traces, ${\left[1\bar{1}00\right]}_{h}$ (A-A) and ${\left[11\bar{2}0\right]}_{h}$ (B-B), correspond to ${\left(11\bar{2}0\right)}_{h}$ and ${\left(1\bar{1}00\right)}_{h}$ interfacial planes between two variants of the O phase with coinciding $130_{o(1)}/\bar{1}30_{o(2)}$ and ${\left(110\right)}_{o}/{\left(1\bar{1}0\right)}_{o}$ planes. (b,c) SAD patterns taken from areas where only one type of interface is present (area A and B on Fig. 15a).

**Fig. 13:**
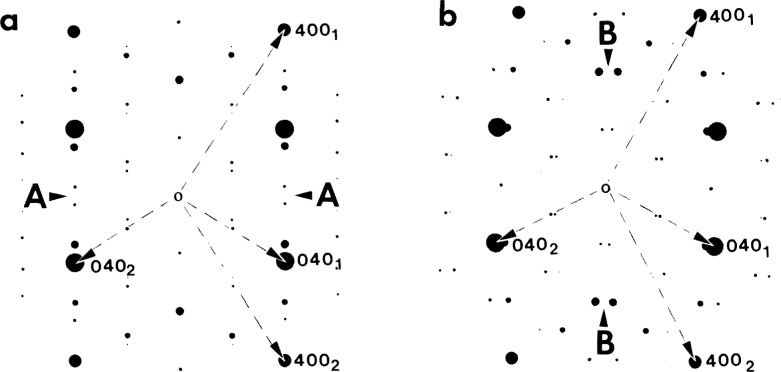
The superimposed [001]_o_ diffraction patterns of two variants of the O phase rotated 120 ° to eaeh other, with a coinciding row of (a) 130_o(1)_ and $\bar{1}30_{o(2)}$ (plane A) and (b) 110_o(1)_ and $1\bar{1}0_{o(2)}$ (plane B) reflections, corresponding to the experimental SAD of Fig. 15b, e, respectively. The kincmatical intensities (size of tbe reflections) are calculated according to the O phase structure parameters in Ref. [19].

The presence of domains of the O phase (as a part of the primary needle substructure) is manifest in the splitting of the DO_19_ reflections and in the complex contrast showing [0001]_h_ directionality as seen in Fig. 11f, i, l. The domains with plate-like morphology can only clearly be seen in the [0001]_h_ zone axis orientation where the domain interfaces are “edge-on”, as shown in Fig. 12a. (In order to obtain maximum contrast, the TEM foil must be slightly off of the exact [0001]_h_ zone axis in order to have a different excitation error and accordingly contrast for different domains.) In Fig. 12 two directions of interface trace, ${\langle 1\bar{1}00\rangle }_{h}$ (A-A) and ${\langle 11\bar{2}0\rangle }_{h}$ (B-B) are observed corresponding to ${\left(11\bar{2}0\right)}_{h}$ and ${\left(1\bar{1}00\right)}_{h}$ interfacial “edge-on” planes. The SAD patterns (Fig. 12b, c) were taken from areas with only one type of interface, A or B in Fig. 12a, respectively. The corresponding SAD patterns are given in Figs. 12b, c. The patterns show splitting and streaking of reflections in directions normal to the interfaces. The SAD patterns from Fig. 12 can be reasonably well explained as belonging to two variants of the O phase, with coinciding (a) ${\left(130\right)}_{o(1)}∥{\left(1\bar{3}0\right)}_{o(2)}$ planes (A-A interfaces) and (b) ${\left(110\right)}_{o(1)}∥{\left(1\bar{1}0\right)}_{o(2)}$ planes (B-B interfaces) as seen in Fig. 13. SAD from a region of bright uniform contrast (upper left side of Fig. 12) shows the hexagonal symmetry of the pattern without the splitting of the peaks. This suggests that this region is untransformed DO_19_ phase.

**Fig. 14:**
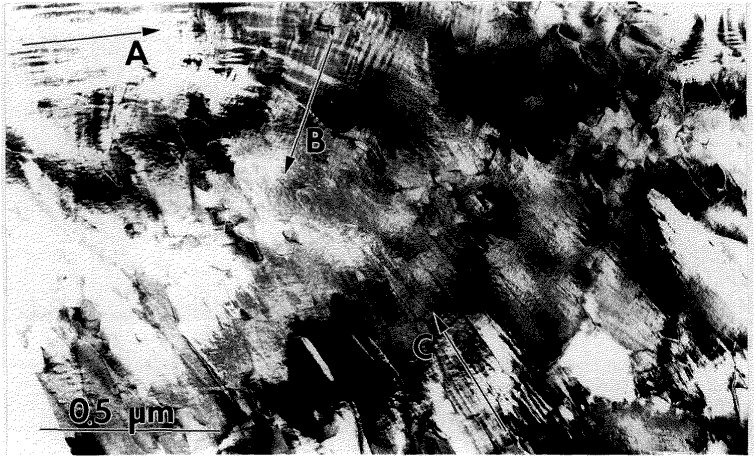
Bright field image of a continuously cooled Alloy 1 specimen, slightly off the [0001] zone axis, where all three orthogonal sets of the O phase variant pairs (shown as A,B and C) rotated with respect to each other by 60 ° arc observed in a single grain.

In a single grain (needle) most often only one orthogonal set of such interfaces (plates) was observed, e.g., ${\left(1\bar{1}00\right)}_{h}$ and ${\left(11\bar{2}0\right)}_{h}$ in Fig. 12. Neighboring grains in the same [0001]_h_ orientation have similar orthogonal sets of plates but rotated 60° or 120°. Occasionally the rotated sets are observed in different locations of the same grain, as is seen in Fig. 14. These sets of interfaces, related to each other by the hexagonal symmetry of the parent phase, belong to other pairs of variants of the orthorhombic phase. The presence of the O phase domains related to each other by the hexagonal symmetry clearly indicates the occurrence of the last step in the transformation path 1; viz., the DO_19_ to O phase transition. As was discussed in [1], the ${\left\{1\bar{1}00\right\}}_{h}$ and ${\left\{11\bar{2}0\right\}}_{h}$ interfaces are SFIs accommodating transformation strains in the hexagonal to orthorhombic symmetry transitions. In this transition the SFIs are always symmetric.

## 5. Decomposition of the Metastable O Phase in Alloy 1 After Prolonged Annealing at 700 °C

**Fig. 15:**
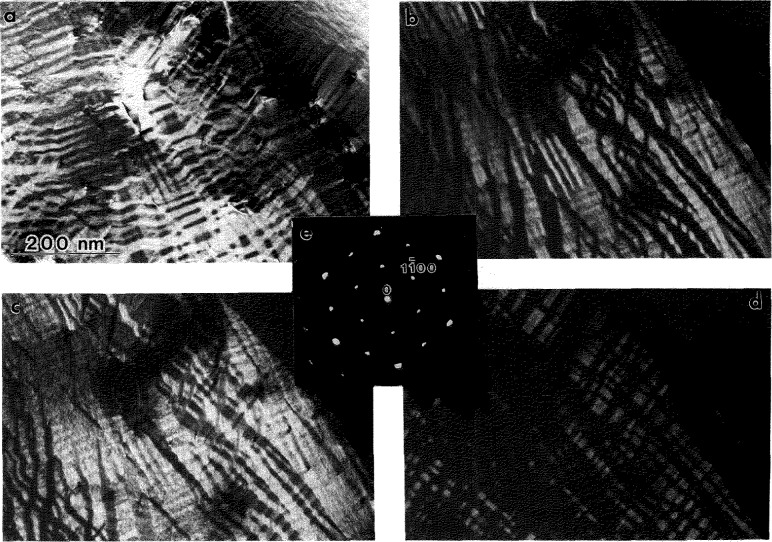
Microstructure of the Alloy 1 after annealing at 700 °C for 26 d. Bright field (a) and three dark field (b–d) images, all slightly different in a TEM foil tilt, show a complex morphology of coherent domains.

Long term annealing of specimens of Alloy 1 at 700 °C for 26 d produces a third level of microstructure finer than that produced during the initial cooling from 1100 °C. The coarser two levels of microstructure (shown in Figs. 8 and 12) are retained during this heat treatment.^2^ The third level is contained within the second level shown in Fig. 12. A typical example of the second and third level microstructure is shown in Fig. 15. The structure within each first level needle remains coherent and preserves the average hexagonal symmetry and the ordering of the transient parent DO_19_ of path 3.1 as the SAD pattern in Fig. 15e shows.

Different dark field images (Fig. 15b, c, d) taken with the same diffuse 1100 reflection but in a slightly different TEM foil orientation reveal fine scale domains and their interfaces (tilting changes the excitation errors for different domains). Therefore the diffuse reflections are in fact clusters of a few reflections very close to each other (additional diffuse intensities from the presence of a high density of interdomain interfaces and lattice strain make it difficult to resolve them). The SAD pattern, Fig. 15e, can be explained by a structure consisting of either three variants of the O phase formed from the DO_19_ phase (as was observed in the specimens cooled from 1100°C, Fig. 12) or coexisting domains of the O and DO_19_ phases.

Because of experimental difficulties related to the similarity of the reciprocal lattices of the phases, the fineness of the domains and possible elastic distortions due to the coherency of interfaces, we were unsuccessful in providing direct TEM evidence of the identity and distribution of the phases. Analysis of the broadening and position of peaks in a neutron diffraction pattern obtained from a specimen similar to that of Fig. 15 has indicated the presence of both O and DO_19_ phases (unpublished research, [19]). Indirect evidence for the phase constitution can be obtained by analyzing the possible orientations for strain-free coherent interfaces which would be expected between the O phase domain variants or between domains of the DO_19_ phase and a variant of the O phase.

For a domain structure of the O phase formed from the DO_19_ phase, as was shown in [1] and confirmed experimentally in Sec. 4.2, the interfaces have locked-in symmetry and have either ${\left\{1\bar{1}00\right\}}_{h}$ or ${\left\{11\bar{2}0\right\}}_{h}$ planes (of the average hexagonal lattice). For contacting domains of the O and DO_19_ phases, the interface orientations depend on the lattice parameters at the temperature of formation, and in general are irrational (non-symmetric). Because the *c* -parameters of the O and DO_19_ phases are similar [19,27], the interfaces are expected to contain the [0001]_h_ direction.

**Fig. 16:**
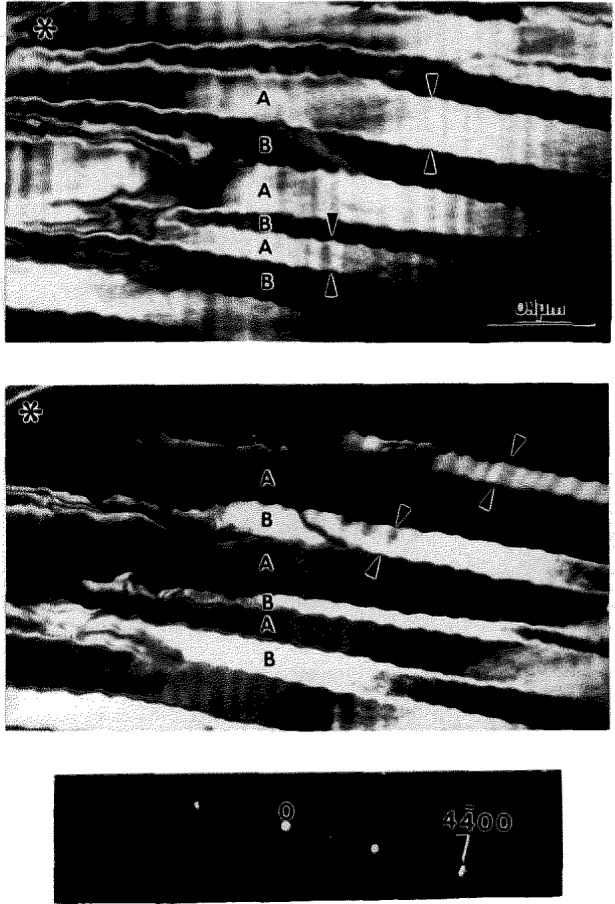
Two dark field images, a and b, taken with the same cluster of reflections ${\left(4\bar{4}00\right)}_{h}$ (e) but different in a small tilt (close to [0001]) show two second level plates, A and B, separated by a zigzag shape interface close to the ${\left(11\bar{2}0\right)}_{h}$ plane. The zigzag shape is formed by two segments of interfaces between the DO_19_ and O phase domains located in neighboring second level plates A and B, The segment planes are irrational orientations.

**Fig. 17:**
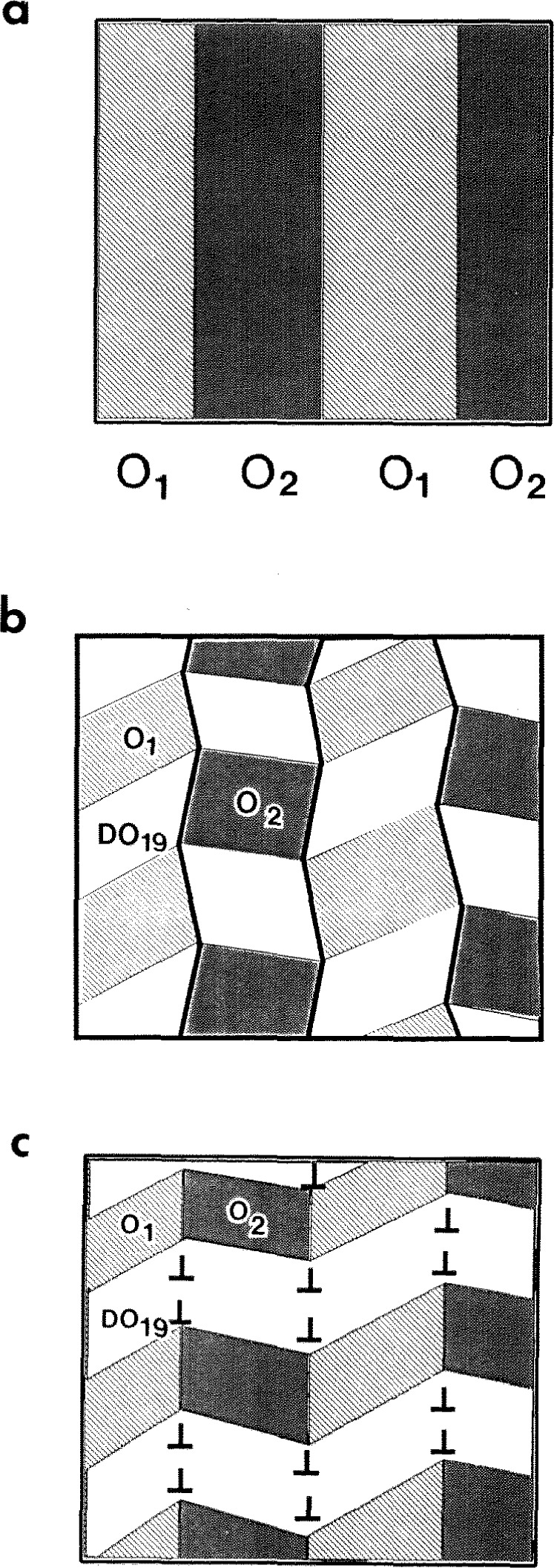
Schematic drawing showing the two-phase (O and DO_19_) domain distribution based on interpretation of the experimental images (Figs. 16, 17). The two-phase structure is formed by reprecipitation of the DO_19_ phase from the initial O phase twinned plates (a). Two morphologies are shown: (b) one with a chess-board distribution of domains surrounded by SFls. (e) another with the DO_19_ phase forms zigzagged ribbons traveling continuously through the second level plates in a modulated manner. The DO_19_ phase ribbon-like domains have internal low angle boundaries (dislocation walls) and faceted SFIs with two variants of the O phase.

Measurement of the directions of the interface traces with respect to the average hexagonal lattice in Fig. 15 are sufficient to establish a significant deviation from the ${\left\{1\bar{1}00\right\}}_{h}$ and ${\left\{11\bar{2}0\right\}}_{h}$ planes despite the relatively large measurement error due to the diffuseness and shortness of the interface segments. We consider this deviation as evidence for the existence of a coexisting two-phase mixture. Such non-symmetric interfaces are often seen lying parallel within a second level plate where the neighboring second level plate also has non-symmetric parallel interfaces but with different orientation. The observation is illustrated in Fig. 16a, b where two dark field images (using the same cluster of reflections, ${\left(4\bar{4}00\right)}_{h}$, but with different small tilts of the TEM foil) show two second level plates, A and B, separately. The interface between them has a zigzag shape but on average is close to the ${\left(11\bar{2}0\right)}_{h}$ plane (Fig. 16c). The zigzag shape is formed by two segments of interfaces between the DO_19_ and O phase domains located in neighboring second level plates A and B. The segments planes also seem to be in irrational orientation. The interpretation of the distribution of domains and phases in the microstructure of Fig. 16 is depicted schematically in Fig. 17a,b. Another plausible two-phase morphology of domains with SFIs is shown schematically in Fig. 17c (and perhaps microstructurally in Fig. 15). In this case the DO_19_ phase forms zigzag ribbons traveling continuously through the second level plates in a modulated manner. The DO_19_ phase ribbon-like domains have internal low angle boundaries (dislocation walls) and faceted SFIs with two variants of the O phase.

Therefore, the variety of interface orientations observed in Fig. 15 is due to the fact that the initial structure consisted of a three variant domain structure of the metastable O phase as seen in Fig. 14 (second level microstructure). Subsequent reformation of the hexagonal DO_19_ phase takes place in the plate-like structure of the O phase (corresponding to A and B plates in Fig. 16). The DO_19_ phase layers can have two equivalent stress-free habit planes for each variant of the O phase. The DO_19_ phase appears as a modulation of plates inside the orthorhombic phase domains. Because the 700 °C annealing results in reprecipitation of the DO_19_ phase, Alloy 1 is believed to be in an equilibrium two-phase field at this temperature.

## 6. Thermodynamics of Phase Formation

**Fig. 18:**
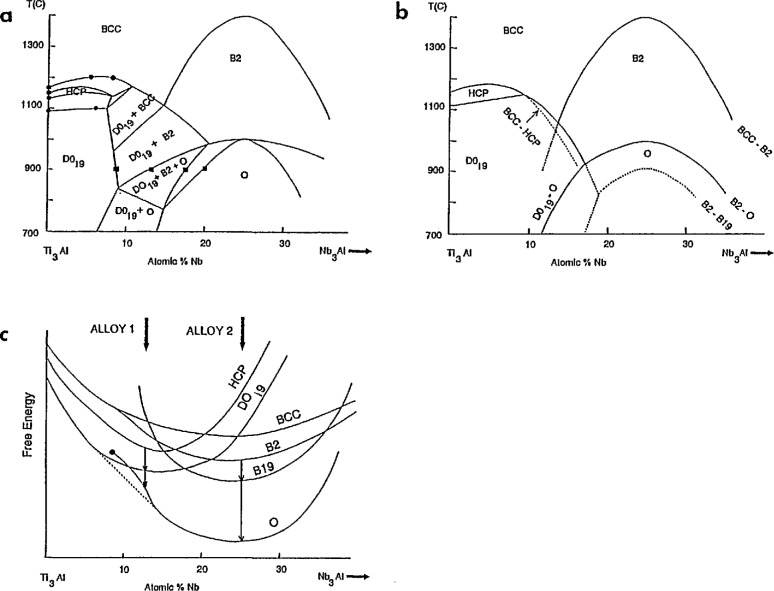
Schematic (a) pseudobinary equilibrium phase diagram, (b) *T*_0_ diagram for partitionless transformations, and (e) 700 °C free energy vs composition curves for the Ti_3_Al-Nb_3_Al section of the Ti-Al-Nb system. The free energy diagram shows the superposition of the BCC (B2) and HCP (DO_19_, B19, and O-phasc) families of phases. For Alloy 2, an intermediate HCP or DO_19_ phase cannot form during partitionless transformation from cubic to the O-phase.

At the present time, neither the phase diagram nor its associated free energy functions are sufficiently well know to permit *a priori* prediction of the *T*_0_ curves for the various BCC/B2 to close-packed transitions in the Ti_3_Al-Nb_3_Al pseudobinary section. These *T*_0_ curves would provide the thermodynamic framework necessary to understand the partitionless transformations observed in the present work; viz., why there is a change in path from 1 to 2 for (Ti,Nb)_3_Al alloys as the Nb content is increased (from that of Alloy 1 to Alloys 2 and 3). However we can use the transformation path results of this paper, some knowledge of the ordering tendencies of BCC and HCP systems, and the limited phase diagram results from other researchers to construct a *self-consistent* pseudobinary section, a *To* diagram, and a 700 °C free energy-composition diagram as shown in Fig. 18. It will be seen that paths including and excluding the intermediate HCP phase are quite reasonable. The construction of the three diagrams was performed concurrently, adjusting curves to be consistent with the details described below.

The free-energy composition diagram (Fig. 18c) should be viewed as a superposition of BCC-based ordering diagram (BCC and B2) and an HCP-based ordering diagram (HCP, DO_19_, B19, and O). The relative heights (energies) of these two subsidiary diagrams have been adjusted to be consistent with the fact that Nb is a beta (BCC) stabilizer, i.e., the HCP phase has a lower free energy at small Nb content than the BCC phase, and conversely at higher Nb content. In fact the intersection of the BCC and HCP free-energy curves (which gives the *T*_0_ composition for the BCC to HCP transition) and the intersection of the BCC and B2 curve (which gives the composition for the BCC to B2 transition) were adjusted to agree with the experimental results of this paper. The individual BCC-based and the HCP-based free energy diagrams are sketched using reasonable assumptions about the ordering tendencies and preferred stoichiometrics for the BCC- and HCP-based phases in this alloy system.

The BCC-based diagram is quite simple and consists of BCC and B2 curves. The BCC→B2 transition is assumed to be second order and thus the B2 free energy curve merges smoothly with that of the parent BCC curve and no two-phase BCC + B2 field exists in the phase diagram. (Thus the *T*_0_ curve and the ordering critical curve are the same). It is reasonable to assume that the composition range of B2 stability exits at intermediate Nb content, probably centered around the Ti_2_AlNb composition for the following reason. The two sublattices (or Wyckoff sites) of the B2 structures of Ti-Al-Nb are known to be preferentially occupied by Ti and a mixture of (Al,Nb) respectively (Ref. 21 in [1]). In the absence of competing non-BCC-based phases, the maximum order is most likely to be centered along the region of the ternary system where the atomic percent of Ti is equal to the sum of the atomic percents of Al and Nb. This region for maximum B2 order and hence for maximum stability intersects the (Ti,Nb)_3_Al section under consideration here at the Ti_2_AlNb composition.^3^ The maximum in the ordering curve is 1400 °C or higher [15].

The free energy curves for the HCP-based phases, A3, B19, DO_19_, and O, are more complex. The HCP→D0_19_, HCP→B19, B19→O and DO_19_→O transitions are all required to be first order transitions under equilibrium conditions [28]. For first order transitions, shapes for free energy vs composition curves that contain end points and concave curvature have been described in detail by Soffa and Laughlin [5] and this shape was used for the orthorhombic ordering in Fig. 18c. The D0_19_ and orthorhombic Ti_2_AlNb phases are assumed to be the equilibrium phases at 700 °C as indicated by the lowest common tangent, giving a tie line that would nearly lie in this pseudobinary section. Generally the tie lines will not lie in the (Ti,Nb)_3_Al section. If, after cooling, an O phase alloy finds itself at a composition and temperature with a concave free energy curve, spontaneous growth of composition fluctuations can occur. If the local composition of a region of that alloy reaches the end point composition, then in that region the ordered phase will spontaneously disorder, in this case, to the DO_19_ phase.

The B19 phase is an AB phase having only two Wyckoff sites with occupancies similar to B2 and would therefore be expected to have maximum stability in the same composition region where the B2 phase has maximum stability, i.e., along the 50% Ti line, which intersects the (Ti,Nb)_3_Al section near the Ti_2_AlNb composition. Thus the B19 free energy curve is centered around this composition as indicated in Fig. 18c. The B19 phase has never been observed as an equilibrium phase in this system and is thus metastable at all temperatures and compositions and does not appear in the phase diagram. Finally the site occupancy of the ordered A_2_BC orthorhombic phase [19] clearly indicates that its compositional range of stability should also be centered around Ti_2_AlNb.

The DO_19_ phase, an A3B phase, is known to have a preference for Al on the B sites and a mixture of (Ti,Nb) on the A sites (Ref. 23 in [1]) in Ti-Al-Nb alloys. Thus stability of this phase with respect to HCP is expected across the entire (Ti,Nb)_3_Al section at 700 °C and hence the free energy curve for DO_19_ is drawn below the HCP. Near the composition Ti_2_AlNb, it is likely that the B19 phase would have a lower free energy than DO_19_ because of the presence of equal amounts of Al and Nb at this composition.

The pseudo-binary phase diagram section (Fig. 18a) was constructed using information on the BCC, HCP and DO_19_ equilibria from the calculated binary Ti-Al and from isothermal sections of Ti-Al-Nb at 1100 and 1200 °C [29]. The positions of phase boundaries between the DO_19_, B2, and O phases at 900 °C were taken from the 900 °C isothermal section of Ref. 7 of [1]. The maximum in the B2 to O transition was placed at 1000 °C according to Ref. 25 of [1]. The remainder of the diagram was sketched to be consistent with Fig. 18 b, c.

The *T*_0_ diagram (Fig. 18b) contains solid curves that correspond to the equilibrium two-phase fields in the phase diagram (Fig. 18a). The ***T***_0_ triple points (intersections of solid curves) correspond to three-phase triangular regions in the phase diagram.^4^ Also indicated are dashed extrapolations of the important BCC to HCP and BCC to B2 curves. A possible location for the ***T***_0_ curve for B2→B19 is also given. These dashed curves only have meaning if the high temperature BCC or B2 parent phase is retained for kinetic reasons during cooling through the higher *T*_0_ curve(s).

We now discuss the observed results using these diagrams. Water-quenched samples of all three alloys in the present work are ordered B2. This rapid quench apparently suppresses the BCC→DO_19_ and the BCC→HCP transformations for Alloy 1 and permits access to the BCC to B2 ordering curve at ~900 °C as shown in Fig. 18b. At a slower cooling rate (~400 K/s), the BCC→DO_19_ transformation is bypassed for the kinetically simpler BCC→HCP transformation that requires only displacive ordering. Once the HCP phase forms, subsequent partitionless transformation to B2 is not possible. The formation of the HCP phase sets the stage for all of the subsequent transformations of Alloy 1. Alloys with higher Nb content can not escape ordering to the B2 at any cooling rate because the ordering temperature is relatively high. Indeed Alloy 2 is B2 at 1200 °C. The presence of the B2 phase sets the stage for the subsequent transformations of Alloys 2 and 3. For Alloys 2 and 3 the transformation BCC→HCP is not possible because of the way the *T*_0_ curve plunges to low temperature.

For simplicity of discussion, the subsequent transformation paths for each alloy are considered as occurring isothermally at 700 °C as indicated by the arrows in Fig. 18c starting from the HCP for Alloy 1 and from the B2 for Alloys 2 and 3 following the above discussion. The sequence for each alloy class undergoes partitionless transformation down a hierarchy of phases with decreasing free energy. One can see that for the alloys near the composition of Alloy 2, a B2→B19→0 path is likely. On the other hand for alloys near the composition of Alloy 1, an HCP→D0_19_→O is likely. Thus we have constructed a set of thermodynamic relationships between the phases that is consistent with the experimentally observed paths for the partitionless transitions.

A later stage of transformation occurs for the low Nb content alloy shown in Fig. 18c that involves long-range diffusion. The concave curvature of the free energy curve indicates that the O phase formed for this composition by partitionless transformation is unstable on a longer time scale with respect to small fluctuations of composition (spinodal decomposition). This kind of process is termed conditional spinodal decomposition [3]. The Nb-poor regions of this decomposition will approach the end point of the O phase free energy curve and will spontaneously disorder (relative to the O phase) to the DO_19_ phase. This process is thought to lead to the third level of domain structure described in Sec. 5.

## 7. Conclusion

During cooling from 1100 °C, the high temperature BCC-based phase of (Ti,Nb)_3_Al alloys decomposes into low temperature orthorhombic phase by two different partitionless paths depending on Nb content. Microstructurally the two paths are differentiated by the substructure of domain boundaries and the number of variants of the orthorhombic phase. For alloys with ~12.5 at% Nb, the transient formation of a hexagonal precursor occurs while at ~25 at% Nb ordering to the B2 precludes the hexagonal phase. In the latter case, defects are found that suggest the transient existence of a B19 phase. However the B19 phase itself was never observed in cooled samples. In the former case formation of the O phase from the ordered DO_19_ was observed along with defects indicating the hexagonal to the DO_19_ ordering. These two different paths are seen as feasible after an examination of subgroup/supergroup relations between the crystal structures of the various phases. The paths are also feasible based on reasonable assumptions regarding the thermodynamic relationships among the free energy curves for the phases involved.

Detailed examination of the interfaces between the rotational domains/variants of the B2 to O phase transformation steps (for Alloys 2 and 3) and the DO_19_ to O phase transformation steps (for Alloy 1) showed they are determined by the minimization of elastic strain energy through the formation of stress-free interfaces with special orientations of twins of the I and II kind. For the Alloy 2 and 3 the twins are often arranged in a self-accommodating polytwin group consisting of three variants of the O phase.

A two-phase modulated microstructure is observed after long term annealing at 700 °C of the Alloy 1. The structure morphology is determined first by a formation of the metastable O phase (by congruent ordering of the DO_19_ phase), and then by reprecipitation of the DO_19_ phase. The thermodynamics underlying the two-phase formation, possibly by a spinodal mechanism, are discussed.

## Figures and Tables

**Table 1:** Experimental dark field visibilities and phase factors (***R***·***g***) for two types of interfaces forming substructure in the O phase in Alloys 2 and 3 (both ***g*** and ***R*** are in the O phase coordinates)

*g* (used in dark field)	002	$\bar{1}10$	$\bar{2}20$	$\bar{1}11$	$\bar{4}40$	400
Type I (curved interfaces)	i	v	i	v	i	i
***R***·***g*** (***R*** = l/2[0 1 0] or	0	1/2	1	1/2	2	0
***R*** = 1/2[1 0 0])	0	−1/2	1	−1/2	0	2
Type II (faceted interfaces)	i	v	v	v	r	i
***R***·***g*** (***R*** = l/4[0 1 2] or	1	1/4	1/2	1/4	1	0
***R*** = l/4[0 1 0])	0	1/4	1/2	1/4	1	0

i –invisible; v–visible; r –residual.

## Footnotes

1 Similar crystallographic planes have been observed in the 2H martensite of Cu-Al alloys. Because the unit cell of the martensite is different from that described here, the indiees of the observed plancs are different [24],

2 The microstructure produced during cooling is more resistant to coarsening and recrystallization as compared to Alloys 2 and 3 under the same annealing conditions [15].

3 The possibility of achicving B2 order decreases as one moves from the TiAl composition to the TiNb composition because of the known positive heat of mixing of the BCC phase in the TiNb binary.

4 The intersection point of two ***T****_o_* eurves neeessarily requires the intersection of a third ***T***_o_, at the same point.
